# Cerebrovascular reactivity and cerebral autoregulation are improved in the supine posture compared to upright in healthy men and women

**DOI:** 10.1371/journal.pone.0229049

**Published:** 2020-03-02

**Authors:** Michelle E. Favre, Valerie Lim, Michael J. Falvo, Jorge M. Serrador

**Affiliations:** 1 Department of Pharmacology, Physiology and Neuroscience, Rutgers Biomedical and Health Sciences, Newark, New Jersey, United States of America; 2 Department of Physical Medicine and Rehabilitation, Rutgers Biomedical and Health Sciences, Newark, New Jersey, United States of America; 3 Department of Veterans Affairs, War Related Illness and Injury Study Center, East Orange, New Jersey, United States of America; 4 Department of Cardiovascular Electronics, National University of Ireland Galway, Galway, Ireland; Ehime University Graduate School of Medicine, JAPAN

## Abstract

Cerebrovascular reactivity and cerebral autoregulation are two major mechanisms that regulate cerebral blood flow. Both mechanisms are typically assessed in either supine or seated postures, but the effects of body position and sex differences remain unclear. This study examined the effects of body posture (supine vs. seated vs. standing) on cerebrovascular reactivity during hyper and hypocapnia and on cerebral autoregulation during spontaneous and slow-paced breathing in healthy men and women using transcranial Doppler ultrasonography of the middle cerebral artery. Results indicated significantly improved cerebrovascular reactivity in the supine compared with seated and standing postures (supine = 3.45±0.67, seated = 2.72±0.53, standing = 2.91±0.62%/mmHg, *P*<0.0167). Similarly, cerebral autoregulatory measures showed significant improvement in the supine posture during slow-paced breathing. Transfer function measures of gain significantly decreased and phase significantly increased in the supine posture compared with seated and standing postures (gain: supine = 1.98±0.56, seated = 2.37±0.53, standing = 2.36±0.71%/mmHg; phase: supine = 59.3±21.7, seated = 39.8±12.5, standing = 36.5±9.7°; all *P*<0.0167). In contrast, body posture had no effect on cerebral autoregulatory measures during spontaneous breathing. Men and women had similar cerebrovascular reactivity and similar cerebral autoregulation during both spontaneous and slow-paced breathing. These data highlight the importance of making comparisons within the same body position to ensure there is not a confounding effect of posture.

## Introduction

Cerebral blood flow is affected by numerous factors including blood pressure, cerebral metabolism, arterial blood gases, the autonomic nervous system, and the vestibular system [1]. Two well-known mechanisms that regulate cerebral blood flow are cerebrovascular reactivity and cerebral autoregulation. Cerebrovascular reactivity represents the ability of the cerebral vessels to dilate and constrict to vasoactive stimuli such as CO_2_ [2]. Cerebral autoregulation is a protective myogenic mechanism that is intrinsic to cerebrovascular smooth muscle, causing dilation and constriction in response to pressure changes to maintain relatively constant cerebral blood flow [3].

Both cerebrovascular reactivity and cerebral autoregulation are typically assessed in either the supine or seated positions, but it remains unclear whether body posture affects results. This knowledge gap is a particularly important consideration because assessing cerebrovascular function is very frequently performed via MRI, which is performed supine. Results when supine may not be representative of the response when upright, which is the posture where the majority of time is spent throughout the day. For example, a patient may show normal cerebrovascular function when supine but impaired when upright, a finding that may be overlooked if only examined when supine. Additionally, the standing posture is often ignored even though individuals may spend a large portion of their day standing and walking. This posture may be important to study in relation to orthostatic tolerance because of the greater orthostatic stress compared to the upright seated posture.

One unique aspect of the upright posture is that there is lower pressure at the brain [4, 5] due to the hydrostatic gradient, which is caused by the force of gravity causing arterial blood pressure to be lowest at the head and greatest at the feet [6]. In the supine posture, there is equal pressure between the brain and the rest of the body. In order to maintain relatively constant cerebral blood flow under lower cerebral perfusion pressure when upright, the cerebral vessels must dilate [6].

Currently, there are mixed results in the literature for the effect of body posture on cerebrovascular function. For example, some studies found significantly improved cerebral autoregulation when supine compared to seated [7] and head-up tilt [8], and improved cerebrovascular reactivity when supine compared to seated [9]. In contrast, other studies found minimal effects of posture on autoregulation [10–12] and cerebrovascular reactivity [13, 14]. Thus, these conflicting findings warrant further investigation.

One possible explanation for mixed results could be due to sex differences in cerebrovascular function. For example, our lab [7, 15, 16] and others [17, 18] have previously found improved autoregulation in women in the upright posture. However, there are contrasting data in the supine posture, where differences are less apparent. To our knowledge, only a few studies have examined sex differences in cerebral autoregulation when supine and the results are mixed [7, 17, 19]. Interestingly, Wang et al. [17] found improved autoregulation in women when upright but reduced when supine, suggesting body posture may affect cerebral autoregulation differently between the sexes. This highlights that both body posture and sex need to be taken into consideration when assessing cerebrovascular function. For example, if women are better at dilating in a state of lower cerebrovascular resistance, such as in the upright posture, one might expect that studies assessing cerebrovascular function in the upright posture will show improved cerebrovascular function in women, while studies in the supine posture may find no improvement in women. Our study focuses on healthy premenopausal women and age-matched men because potential confounding vascular factors such as menopausal state, tobacco use, and diabetes may affect the cerebrovascular response.

We hypothesized that both cerebrovascular reactivity and cerebral autoregulation would be enhanced in the supine posture compared to the seated and standing postures. We also hypothesized that women would show improved autoregulation compared to men when upright, but not when supine.

## Materials and methods

This study was approved by the Rutgers Health Sciences Institutional Review Board Newark and was conducted under the guidelines established by the Declaration of Helsinki. All participants were provided with written and verbal explanations of study procedures in detail before providing their written informed consent.

### Participants

Twenty-three healthy individuals (11 women, 12 men) without a history of cardiovascular, cerebrovascular, or other diseases were enrolled. One woman and one man were excluded from the analysis due to caffeine ingestion or a poor middle cerebral artery Doppler signal. Men and women were similar in age (men: 28 ± 9 years, range 18–47; women: 29 ± 9 years, range 23–54). One participant of each sex was over 36 years and inclusion of these participants did not affect results.

Participants refrained from cold or headache medication for at least twenty-four hours, avoided exercise, caffeine, and alcohol for at least twelve hours, and refrained from food and drink (except water) for at least two hours before study procedures. Participants were in their usual state of health and slept for at least six hours (7.45 ± 1.0 hours) the night before the study.

Cerebrovascular reactivity and cerebral autoregulation were unaffected by menstrual cycle [16]. The cerebrovascular response to paced, deep breathing was unaffected by menstrual cycle and oral contraceptives [20], so time of cycle and contraceptive use was not controlled. Three women were on monophasic, combined oral contraceptive pills and all were measured during the hormone phase of the pill. Seven women had natural menstrual cycles. Three women were tested during the follicular phase (range: days 6–14) and four were tested in the luteal phase (range: days 24–35). All women were confirmed not pregnant immediately prior to laboratory testing. Laboratory conditions were kept between 20.0–22.5°C.

### Study design

Participants performed cerebrovascular reactivity and slow-paced breathing tests three times, once each in the following postures: 1) supine, 2) seated, and 3) standing. The order of body posture was randomized and counterbalanced across participants. All participants began with the slow breathing test. Once the physiological measurements returned to their baseline levels, the cerebrovascular reactivity test was performed. Participants remained in the same body position and were allowed to move their legs and talk as needed. Since passive standing may result in syncope, participants were screened for a history of fainting and closely watched for pre-syncopal symptoms while standing. No participant had a history of fainting or developed pre-syncopal symptoms while standing during the tests or during the break between tests. Participants waited ten minutes between each body posture before beginning the slow breathing and cerebrovascular reactivity test in the next body posture.

### Study procedures

#### Cerebrovascular reactivity test

Participants were instructed to breathe spontaneously for two minutes before breathing a hypercapnic gas mixture (5% CO_2_, 21% O_2_ and balanced N_2_) through an oxygen mask for two minutes. Immediately after hypercapnia, participants were instructed to mildly hyperventilate for two minutes to induce hypocapnia. Participants were paced by study staff to achieve an end-tidal CO_2_ ~10 mmHg below their baseline end-tidal CO_2_ as previously described [15, 16].

#### Slow-paced breathing test

Participants were instructed to breathe spontaneously for five minutes followed by breathing to a metronome at a rate of six breaths/minute (five second inhalation, five second exhalation) for an additional five minutes.

#### Physiological measurements

Transcranial Doppler ultrasonography was used to measure continuous cerebral blood flow velocity in the right middle cerebral artery (MCA) with a 2-MHz probe (MultiDop X4, DWL, Germany). The probe was fixed in place for the duration of the study using a custom-made probe holder and Velcro headband. The probe was positioned over the trans-temporal window as previously described [21] to ensure correct insonation of the MCA. Beat-by-beat blood pressure was measured using non-invasive finger plethysmography (Finapres, Ohmeda, Netherlands and/or Finometer Pro, Netherlands) on participants’ non-dominant hand, positioned in an arm sling. If the hand was not positioned at heart level, mean arterial pressure was corrected for by multiplying the hydrostatic gradient factor 0.7355 mmHg/cm [22] by the distance between the heart and finger cuff. A sphygmomanometer (Omron Healthcare, IL, USA) on the upper arm was used to confirm accurate blood pressure recordings from the finger cuff. Cerebrovascular resistance was calculated as mean arterial pressure divided by cerebral flow velocity in the supine position. Since the head is positioned above the heart in the upright posture, cerebrovascular resistance and cerebral perfusion pressure in the seated and standing positions were corrected for by multiplying the vertical distance between the heart and MCA probe by the hydrostatic gradient factor 0.7355 mmHg/cm [22]. For example, if the mean arterial pressure was 80 mmHg and there was a distance of 25 cm between the MCA probe and the heart, the cerebral perfusion pressure was estimated to be 61.6 mmHg (80 –(25 * 0.7355)). Breath-by-breath end-tidal CO_2_ was measured using a nasal cannula and capnograph (Puritan-Bennett, MA, USA). Continuous heart rate was measured using a three-lead electrocardiogram (Welch Allyn, OR, USA).

### Data analysis

All physiological measurements of arterial blood pressure, middle cerebral flow velocity, heart rate and end tidal CO_2_ were sampled simultaneously in real-time at 1,000 Hz using Powerlab data-acquisition software (ADInstruments, Colorado Springs, CO). Before analysis, all signals were visually inspected and any beats with artifacts or signal loss were removed and linearly interpolated using a custom written MATLAB script (MathWorks, Natick, MA).

#### Cerebrovascular reactivity

Normalized middle cerebral flow velocity (MCAv) was plotted against the corresponding end-tidal CO_2_ for each breath. The slope between the entire range of end-tidal CO_2_ (baseline, hypercapnia and hypocapnia) and MCAv was used as an index of overall reactivity. To isolate the dilatory component of cerebrovascular reactivity, the slope between MCAv and end-tidal CO_2_ from baseline to hypercapnia was used. The vasoconstrictive component of cerebrovascular reactivity was measured as the slope between MCAv and end-tidal CO_2_ from baseline to hypocapnia.

#### Cerebral autoregulation

We examined cerebral autoregulation during both spontaneous and 0.1 Hz induced oscillations in blood pressure and normalized MCA velocity induced during slow-paced breathing. We assessed transfer function gain, phase, and coherence in the very low frequency (0.02–0.07 Hz) and low frequency (0.07–0.20 Hz) ranges using the Cerebral Autoregulation Research Network’s (CARNet) MATLAB script [23].

Transfer function parameters of gain, phase and coherence are detailed elsewhere [23–25]. Briefly, a lower gain represents improved autoregulation by greater cerebral vessel dampening of blood pressure, reducing the transfer of pressure into cerebral blood flow [25]. Positive phase shifts signify improved autoregulation by active cerebral vessel dilation and constriction in response to changes in blood pressure [25]. A coherence closer to 0 is described as improved autoregulation by having less linearity between blood pressure and cerebral blood flow [25].

CARNet recommends using estimates of gain and phase in conditions were there is sufficient coherence (>0.5) between blood pressure and cerebral flow velocity [23]. Due to low coherence in the very low frequency range, estimates of gain and phase from three women during spontaneous breathing in the supine position were not included in the analysis. Additionally, gain estimates from two women and three men while supine, one man when seated, and one woman and one man while standing during slow breathing were not included in the analysis. As per CARNet recommendations, negative phase values in the very low frequency range were removed from the data, [23] which caused us to exclude phase estimates from three women and three men while supine, and from one woman and one man while both seated and standing during slow breathing.

### Statistics

The data were analyzed using SPSS statistical software (IBM, Version 24 Amok, NY: IBM Corp). Our sample size was estimated based off of the cerebrovascular reactivity results by Regan et al. [14], which found a reactivity while supine of 4.09 ± 1.28 vs. seated of 3.1 ± 0.35%/mmHg. Using G*Power 3.1.9.2 with an alpha set at 0.05, power of 80%, and two-tailed paired t-test, we needed a minimum of thirteen participants to investigate postural effects. Even though we had sufficient power, non-parametric statistical analyses were performed on our data to reduce the risk of type I error in our small sample size. Data from men and women were combined to determine the effect of body posture using Friedman’s test. If there was a significant effect of body posture, post-hoc paired samples Wilcoxon signed-rank tests with Bonferroni correction (α adjusted significance threshold: *P* = 0.0167) were used to determine which postures significantly differed. To determine the effect of sex between men and women within each posture, two-tailed independent samples Mann-Whitney tests were used on data between men and women in the supine, seated and standing postures. The threshold for statistical significance for the main effects of posture and sex were set at *P* <0.05. The threshold for significance of post-hoc tests for the effect of body posture was set at *P* < 0.0167 to account for the three postures (α = 0.05/3). All data are represented as mean ± standard deviation. Data are publicly available at the following link: dx.doi.org/10.17504/protocols.io.bb2siqee.

## Results

### Participant characteristics

**Fig 1:**
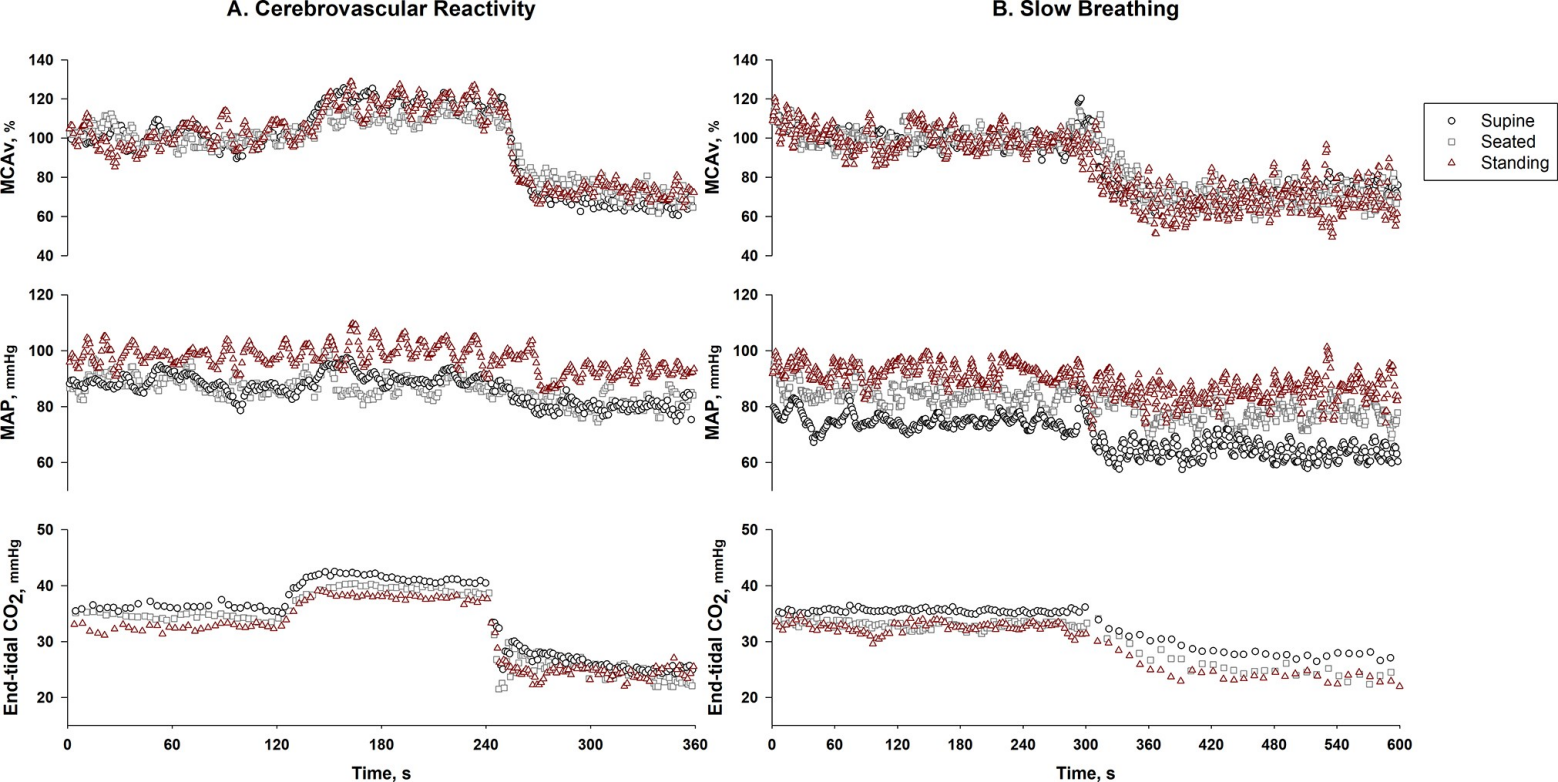
Representative breath-by-breath cerebrovascular CO_2_ reactivity (A) and slow breathing (B) data from an individual across supine, seated, and standing postures. Black open circles represent supine posture. Gray open squares represent seated posture. Red open triangles represent standing posture. The top panel is normalized middle cerebral artery velocity (MCAv). The middle panel is mean arterial pressure (MAP), and the bottom panel is end-tidal CO_2_.

Participant characteristics grouped by sex are reported in Table 1. Representative breath-by-breath cerebrovascular reactivity and slow breathing data from an individual across the three postures are shown in Fig 1A and 1B, respectively.

**Table 1:** Participant characteristics.

	Women	Men	*P* value
***n***	10	11	-
**Age, years**	29 ± 9	28 ± 9	0.809
**Height, cm**	165.3 ± 5.5 *	176.2 ± 9.0	0.004
**Weight, kg**	64.2 ± 5.7 *	76.9 ± 11.9	0.010
**BMI**	23.6 ± 2.6	24.8 ± 3.7	0.468

Values are means ± SD.

* *P* < 0.05 vs. men.

### MCAv cerebrovascular CO_2_ reactivity

**Fig 2:**
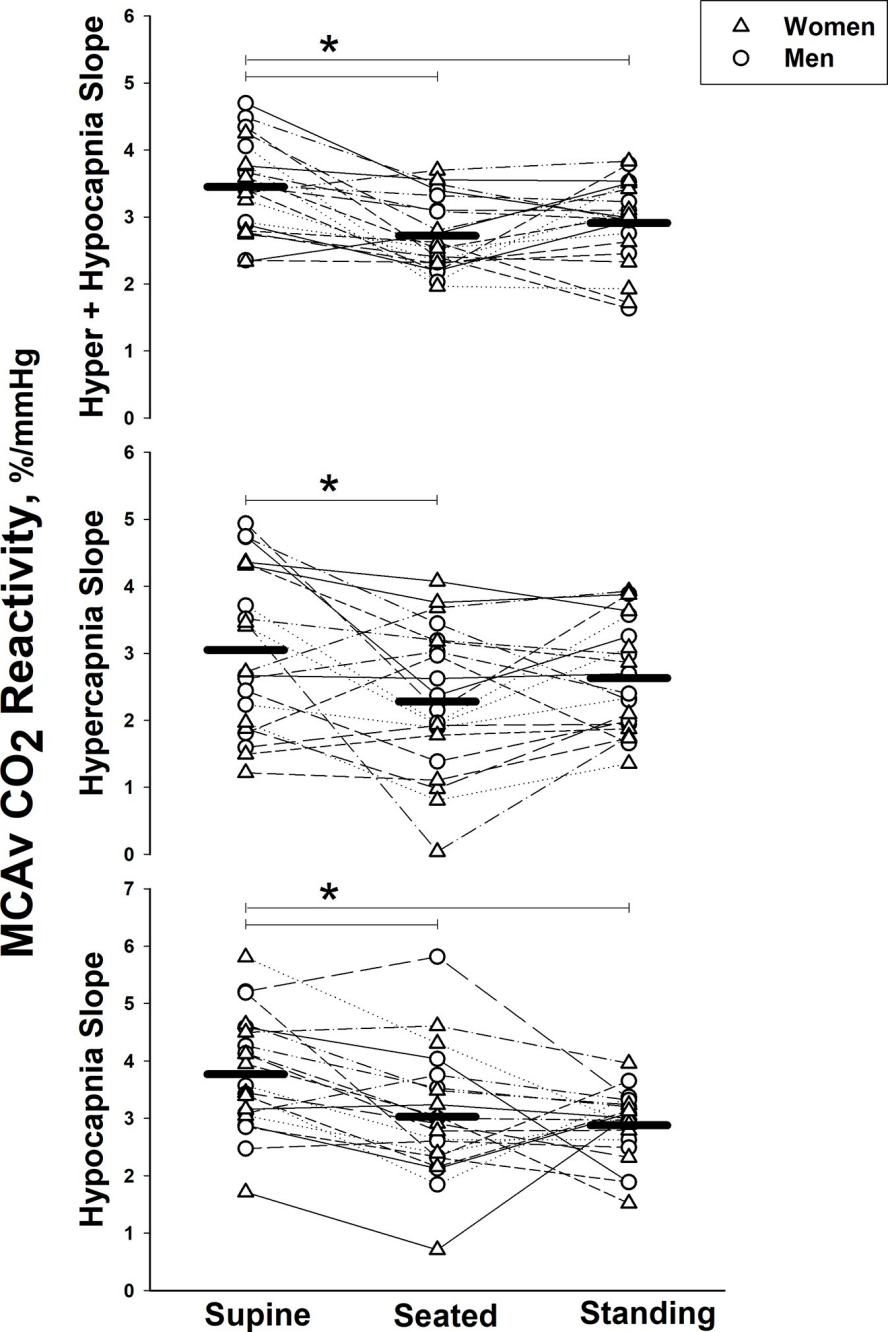
Effect of body posture on cerebrovascular CO_2_ reactivity. The slope across the entire CO_2_ range during hypercapnia and hypocapnia (top), the vasodilatory slope during hypercapnia (middle), and the vasoconstrictive slope during hypocapnia (bottom) in the middle cerebral artery in women (*n* = 10, triangles) and men (*n* = 11, circles) across supine, seated and standing body postures. Lines with symbols represent individual data and solid lines represent group means. * indicates *P* < 0.0167 between postures.

**Fig 3:**
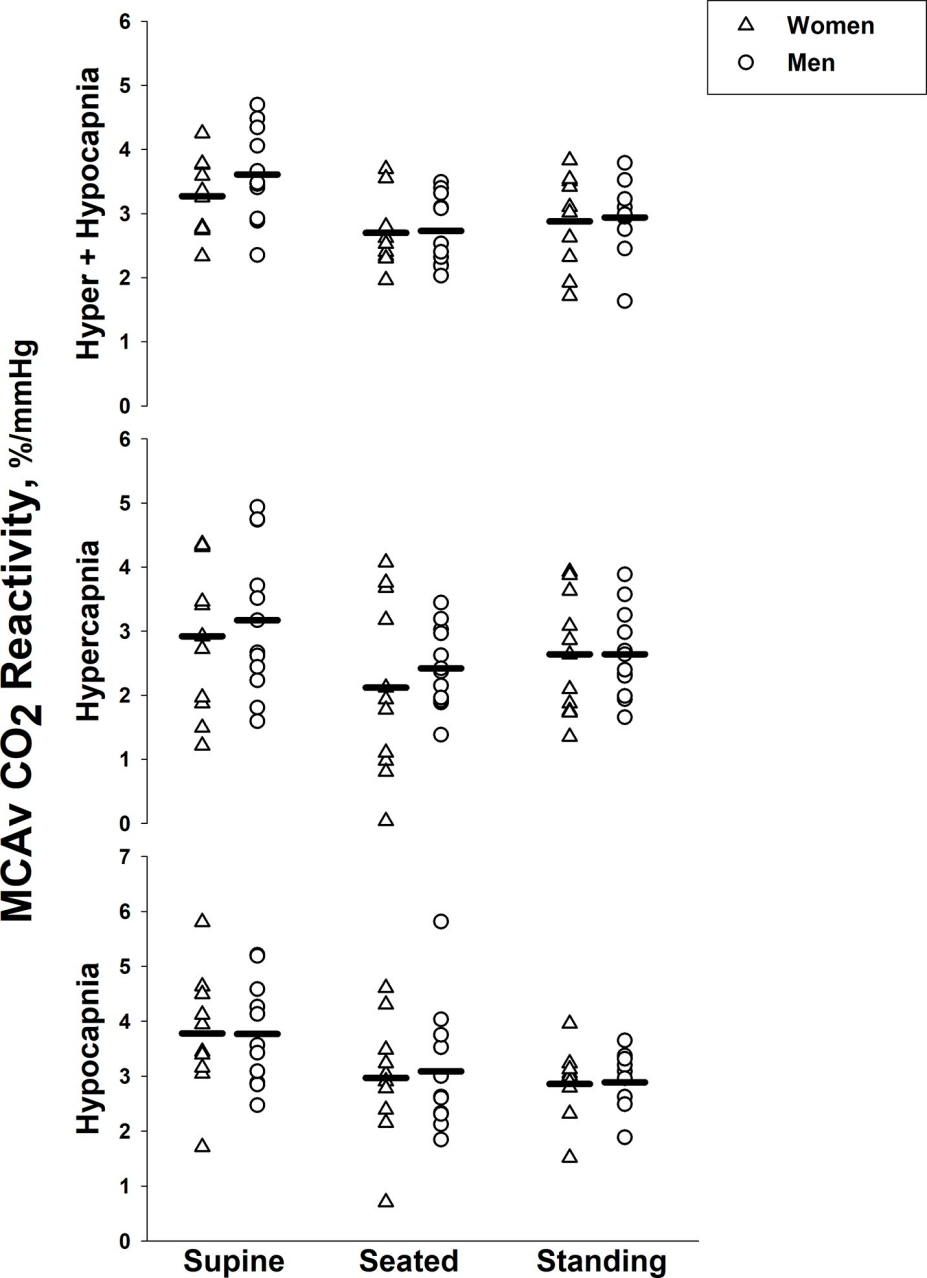
Effect of sex on cerebrovascular CO_2_ reactivity. The slope across the entire CO_2_ range during hypercapnia and hypocapnia (top), the vasodilatory slope during hypercapnia (middle), and the vasoconstrictive slope during hypocapnia (bottom) in the middle cerebral artery in women (*n* = 10, triangles) and men (*n* = 11, circles) across supine, seated and standing body postures. Symbols represent individual male and female data points and solid lines represent group means.

When combining men and women, cerebrovascular reactivity indices were greater when supine compared with both seated and standing postures (all *P* < 0.0167 indicating a significant effect after Bonferroni correction), with the exception of hypercapnic cerebrovascular reactivity when standing (*P* = 0.033; Fig 2). There were no differences between seated and standing postures. There were no sex differences in any indices of MCAv cerebrovascular reactivity (Fig 3).

### Supine, seated and standing hemodynamics during the cerebrovascular CO_2_ reactivity test

Resting hemodynamics in men and women across the three body postures during normocapnia, hypercapnia and hypocapnia are reported in Table 2. Briefly, diastolic blood pressure significantly increased and cerebral perfusion pressure (CPP), MCAv, MCA cerebrovascular resistance, and end-tidal CO_2_ all significantly decreased when standing. There were minimal sex differences.

**Table 2:** Supine, seated, and standing hemodynamics during the cerebrovascular CO_2_ reactivity test.

		Supine	Seated	Standing
**MAP, mmHg**				
*Baseline*	**men**	78.4 ± 7.2	82.4 ± 10.1	82.0 ± 9.8
	**women**	77.8 ± 5.4	78.1 ± 8.1	78.2 ± 8.7
*Hypercapnia*	**men**	79.1 ± 7.8	82.0 ± 10.7	84.0 ± 9.7
	**women**	77.5 ± 5.9	78.5 ± 7.8	81.1 ± 8.6
*Hypocapnia*	**men**	76.2 ± 7.3	80.2 ± 8.4	81.7 ± 9.3
	**women**	73.7 ± 5.1	75.3 ± 6.8	78.3 ± 8.0
**SBP, mmHg**				
*Baseline*	**men**	111.6 ± 9.1	119.9 ± 11.0	114.2 ± 14.1‡
	**women**	113.6 ± 5.1	112.6 ± 7.5*	107.5 ± 8.8‡
*Hypercapnia*	**men**	114.9 ± 8.9	119.9 ± 10.4	117.2 ± 13.8
	**women**	112.9 ± 5.3	112.3 ± 8.1*	111.8 ± 10.1
*Hypocapnia*	**men**	111.8 ± 8.8	119.8 ± 9.8	115.2 ± 12.9
	**women**	108.6 ± 5.9	109.7 ± 6.6*	109.0 ± 8.7
**DBP, mmHg**				
*Baseline*	**men**	63.6 ± 6.9	66.5 ± 9.2	68.7 ± 8.6†‡
	**women**	60.1 ± 5.0	61.0 ± 6.9	64.0 ± 7.7†‡
*Hypercapnia*	**men**	63.8 ± 7.4	65.7 ± 9.6	70.0 ± 8.3†‡
	**women**	59.8 ± 5.2	61.1 ± 6.5	66.2 ± 7.6†‡
*Hypocapnia*	**men**	60.8 ± 7.1	63.6 ± 7.7	67.7 ± 8.3†‡
	**women**	56.2 ± 5.3	58.0 ± 6.2	63.4 ± 7.0†‡
**Mean MCAv, cm/s**				
*Baseline*	**men**	73.6 ± 20.7	65.3 ± 15.7†	59.4 ± 17.1†‡
	**women**	84.9 ± 16.4	80.7 ± 16.9*†	71.7 ± 15.2†‡
*Hypercapnia*	**men**	87.8 ± 20.5	75.2 ± 18.6†	70.8 ± 18.2†‡
	**women**	98.2 ± 23.0	90.1 ± 22.9†	85.3 ± 21.9†‡
*Hypocapnia*	**men**	57.5 ± 14.0	52.3 ± 10.5	47.7 ± 9.4†‡
	**women**	65.7 ± 14.6	65.1 ± 16.0*	58.0 ± 17.5†‡
**Mean CPP, mmHg**				
*Baseline*	**men**	78.4 ± 7.2	61.3 ± 9.6†	60.9 ± 8.8†
	**women**	77.8 ± 5.4	57.0 ± 8.6†	57.0 ± 8.8†
*Hypercapnia*	**men**	79.1 ± 7.8	60.9 ± 10.2†	62.8 ± 8.3†
	**women**	77.5 ± 5.9	57.4 ± 8.4†	60.0 ± 8.9†
*Hypocapnia*	**men**	76.2 ± 7.3	59.1 ± 8.1†	60.5 ± 7.9†
	**women**	73.7 ± 5.1	54.2 ± 7.4†	57.2 ± 9.0†
**Mean MCA Cerebrovascular Resistance, mmHg/cm/s**				
*Baseline*	**men**	1.14 ± 0.32	0.98 ± 0.25†	1.10 ± 0.32†‡
	**women**	0.95 ± 0.21	0.73 ± 0.17*†	0.82 ± 0.18*†‡
*Hypercapnia*	**men**	0.95 ± 0.28	0.85 ± 0.22†	0.94 ± 0.25‡
	**women**	0.83 ± 0.20	0.66 ± 0.15*†	0.73 ± 0.17‡
*Hypocapnia*	**men**	1.39 ± 0.35	1.17 ± 0.28†	1.31 ± 0.25†‡
	**women**	1.17 ± 0.28	0.88 ± 0.24*†	1.04 ± 0.28*†‡
**End-tidal CO**_**2**_**, mmHg**				
*Baseline*	**men**	39.7 ± 4.1	37.2 ± 3.4†	34.0 ± 5.6†‡
	**women**	37.1 ± 2.9	36.4 ± 3.0†	33.5 ± 3.9†‡
*Hypercapnia*	**men**	45.9 ± 3.3	43.6 ± 3.8†	41.5 ± 4.5†‡
	**women**	42.7 ± 3.4	41.4 ± 2.3†	40.3 ± 3.6†‡
*Hypocapnia*	**men**	32.8 ± 3.0	28.6 ± 3.3†	26.0 ± 4.0†‡
	**women**	30.1 ± 3.3	29.0 ± 3.5†	24.8 ± 4.7†‡

Values are means ± SD. MAP, mean arterial pressure; SBP, systolic blood pressure; DBP, diastolic blood pressure; MCAv, middle cerebral artery velocity; CPP, cerebral perfusion pressure; MCA, middle cerebral artery; women *n* = 10, men *n* = 11.

* main effect of sex; *P <* 0.05 vs men;

^†^ *P <* 0.0167 vs. supine;

^‡^ *P <* 0.0167 vs seated.

### Cerebral autoregulation during slow breathing

**Fig 4:**
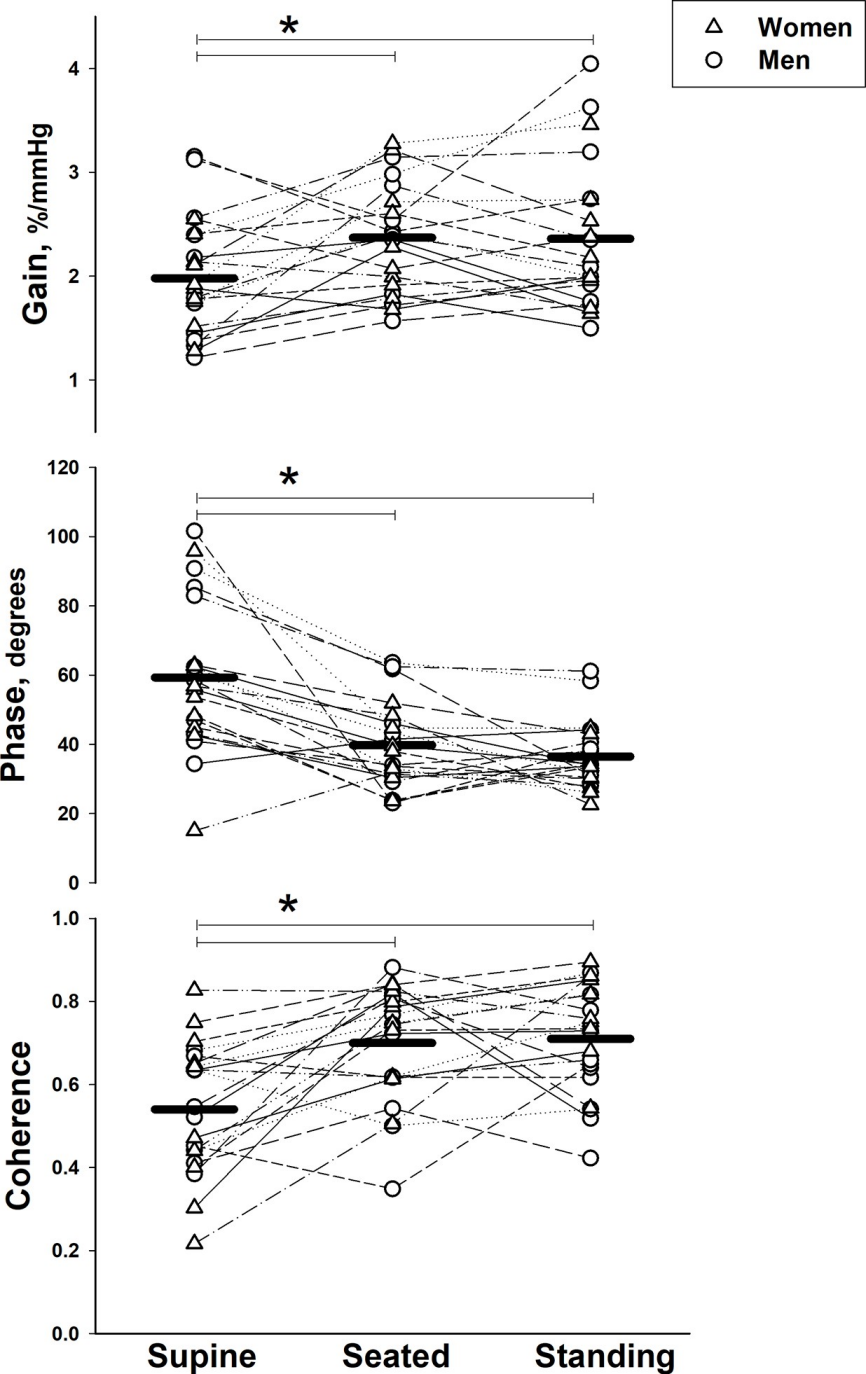
Effect of body posture on cerebral autoregulation during slow breathing. Gain (top), phase (middle), and coherence (bottom) in the middle cerebral artery in the low frequency (0.07–0.20 Hz) range in and women (*n* = 10, triangles) and men (*n* = 11, circles) across supine, seated and standing body postures. Symbols represent individual data points and solid lines represent group means. * indicates *P* < 0.0167 between postures.

During slow breathing, we found improved autoregulatory measures (i.e., lower gain, higher phase and lower coherence) in the low frequency range when supine compared with seated and standing (Fig 4 and Table 3). In the very low frequency range, coherence was significantly higher compared with seated and standing, but there were no differences in gain or phase. The mean amplitude of oscillations in middle cerebral artery velocity and mean arterial pressure during slow breathing were significantly lower when supine compared to seated and standing conditions (Table 4). There was no difference in the variability of end-tidal CO_2_ across the postures (Table 4). There were no differences in any autoregulatory or hemodynamic measures between seated and standing postures.

**Table 3:** Cerebral autoregulation during slow breathing.

		Supine	Seated	Standing
**Gain, %/mmHg**				
*VLF*	**men**	1.31 ± 0.46	1.17 ± 0.25	1.32 ± 0.32
	**women**	1.77 ± 0.54	1.23 ± 0.32	1.35 ± 0.40
*LF*	**men**	2.03 ± 0.70	2.39 ± 0.51†	2.45 ± 0.84†
	**women**	1.93 ± 0.39	2.35 ± 0.57†	2.26 ± 0.55†
**Phase, degrees**				
*VLF*	**men**	94.0 ± 37.6	77.7 ± 15.8	68.9 ± 15.6
	**women**	81.5 ± 45.1	75.5 ± 23.4	74.2 ± 19.1
*LF*	**men**	64.3 ± 22.7	41.8 ± 15.3†	40.0 ± 10.8†
	**women**	53.8 ± 20.2	37.5 ± 8.7†	32.6 ± 7.0†
**Coherence**				
*VLF*	**men**	0.31 ± 0.13	0.46 ± 0.20†	0.42 ± 0.15†
	**women**	0.24 ± 0.12	0.38 ± 0.12†	0.42 ± 0.12†
*LF*	**men**	0.55 ± 0.11	0.67 ± 0.16†	0.66 ± 0.13†
	**women**	0.54 ± 0.20	0.73 ± 0.11†	0.78 ± 0.11*†
**MAP Power, mmHg**^**2**^				
*VLF*	**men**	3.5 ± 2.3	6.1 ± 3.7†	6.4 ± 3.9†
	**women**	1.6 ± 0.8*	3.4 ± 1.3†	4.6 ± 2.8†
*LF*	**men**	5.3 ± 2.6	11.2 ± 6.1†	12.7 ± 8.1†
	**women**	4.9 ± 2.3	8.1 ± 5.0†	9.6 ± 4.0†
**MCAv Power, %**^**2**^				
*VLF*	**men**	10.1 ± 5.9	15.5 ± 13.7	16.4 ± 13.4
	**women**	12.2 ± 9.1	9.1 ± 3.3	14.9 ± 11.3
*LF*	**men**	21.4 ± 18.0	75.0 ± 80.9†	97.8 ± 91.6†
	**women**	15.4 ± 8.8	39.3 ± 26.5†	60.2 ± 51.2†
**MAP, mmHg**				
	**men**	74.3 ± 7.5	75.5 ± 9.3	74.3 ± 8.8
	**women**	68.9 ± 8.2	72.9 ± 7.9	73.2 ± 8.8
**MCAv, cm/s**				
	**men**	55.3 ± 15.3	51.1 ± 15.5†	48.6 ± 13.6†
	**women**	67.6 ± 20.2	64.7 ± 19.3†	60.7 ± 19.4†
**MCAv, %**				
	**men**	100	92.2 ± 8.8†	88.4 ± 10.5†
	**women**	100	95.9 ± 7.3†	89.5 ± 5.3†
**MCA CPP, mmHg**				
	**men**	74.3 ± 7.5	48.1 ± 11.1†	46.9 ± 10.4†
	**women**	68.9 ± 8.2	45.1 ± 10.7†	45.5 ± 11.3†
**MCA Cerebrovascular Resistance, mmHg/cm/s**				
	**men**	1.42 ± 0.35	1.00 ± 0.30†	1.02 ± 0.29†
	**women**	1.09 ± 0.32	0.74 ± 0.25†	0.79 ± 0.27†
**End-tidal CO**_**2**_**, mmHg**				
	**men**	37.0 ± 5.4	32.5 ± 6.1†	31.4 ± 6.1†
	**women**	34.8 ± 4.8	33.0 ± 4.7†	31.7 ± 5.4†

Values are means ± SD. VLF, very low frequency range (0.02–0.07 Hz); LF, low frequency range (0.07–0.20 Hz); MAP, mean arterial pressure; MCAv, middle cerebral artery velocity; women *n =* 10, men *n =* 11, except gain in the very low frequency range when supine (women *n =* 8, men *n =* 8), when seated (women *n =* 10, men *n =* 10) and standing (women *n =* 9, men *n =* 10), and phase in the very low frequency range when supine (women *n =* 7, men *n =* 8), and when seated and standing (women *n =* 9, men *n =* 10).

* main effect of sex; *P <* 0.05 vs. men;

^†^ *P <* 0.0167 vs. supine;

^‡^ *P <* 0.0167 vs. seated.

**Table 4:** Mean amplitude of oscillations in MCAv and MAP, and variability in end-tidal CO_2_ across 10s cycles of slow breathing.

		Supine	Seated	Standing
Mean Amplitude of Oscillations Within 10s Cycles				
**MCAv, %**				
	**men**	± 6.3 ± 1.9	± 10.3 ± 3.5†	± 11.6 ± 5.2†
	**women**	± 6.4 ± 1.4	± 8.6 ± 1.7†	± 10.2 ± 2.2†
**MAP, mmHg**				
	**men**	± 3.9 ± 1.0	± 5.6 ± 1.5†	± 5.9 ± 1.5†
	**women**	± 3.7 ± 0.8	± 4.8 ± 1.2†	± 5.6 ± 1.1†
Variability of Change Between 10s Cycles				
**End-tidal CO**_**2**_**, mmHg**				
	**men**	± 1.6 ± 0.8	± 1.8 ± 0.6	± 1.5 ± 0.6
	**women**	± 1.3 ± 0.4	± 1.6 ± 0.8	± 1.6 ± 0.7

Values are means ± SD. MCAv, middle cerebral artery velocity; MAP, mean arterial pressure; women *n =* 10, men *n =* 11.

^†^ *P <* 0.0167 vs. supine

**Fig 5:**
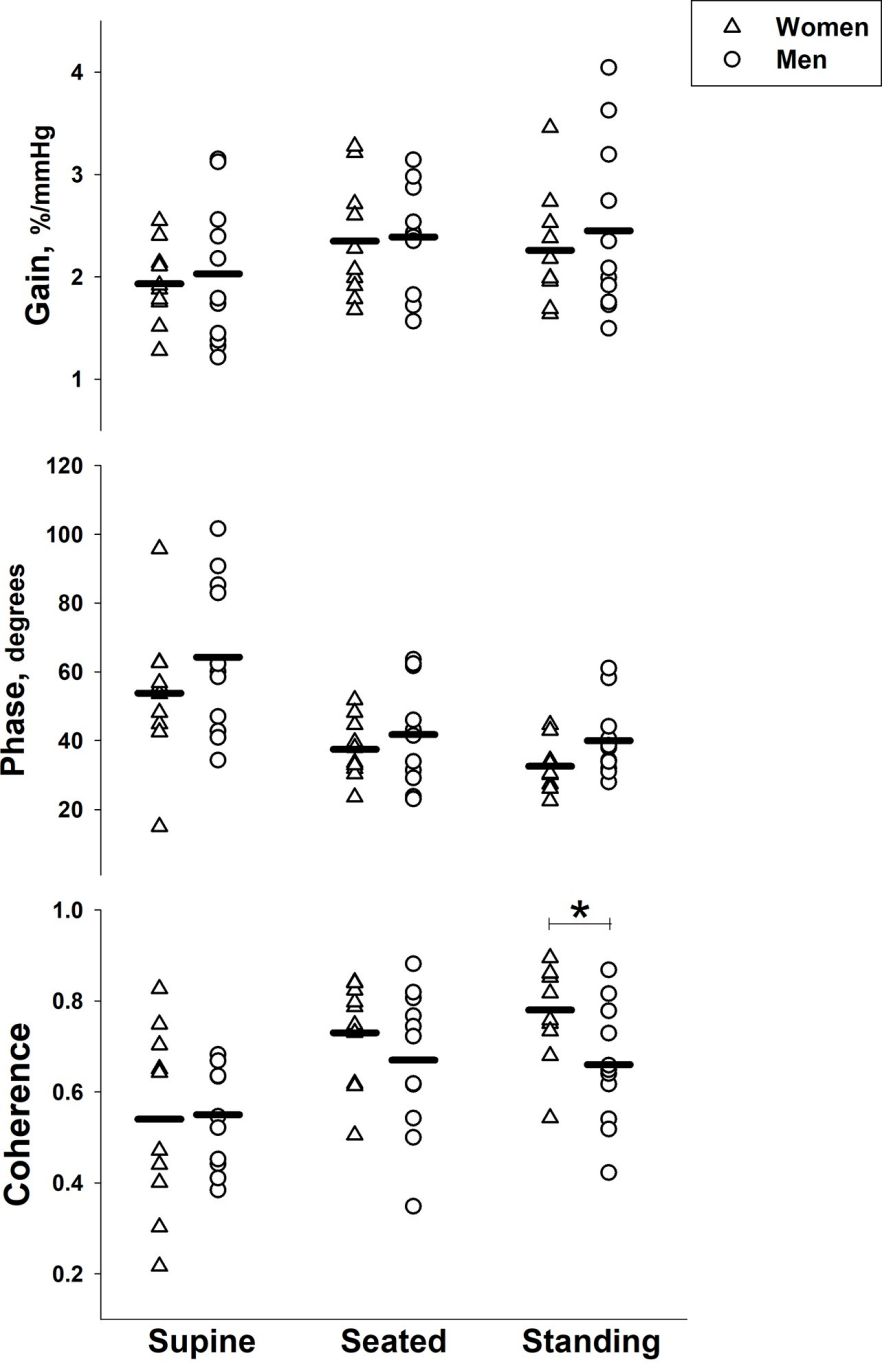
Effect of sex on cerebral autoregulation during slow breathing. Gain (top), phase (middle), and coherence (bottom) in the middle cerebral artery in the low frequency (0.07–0.20 Hz) range in women (*n* = 10, triangles) and men (*n* = 11, circles) across supine, seated and standing body postures. Symbols represent individual male and female data points and solid lines represent group means. * indicates *P* < 0.05 between men and women.

There were no sex differences in any measure of cerebral autoregulation except significantly higher coherence in women in the low frequency (LF) range when standing (*P =* 0.036; Fig 5 and Table 3). There were no sex differences in any cerebral hemodynamic measure.

### Cerebral autoregulation during spontaneous breathing

During spontaneous breathing, there were no differences in cerebral autoregulatory measures across body postures in both the VLF and LF ranges with the exception of significantly lower coherence in the LF range when supine compared to both seated (*P* = 0.003) and standing (*P* < 0.0001) positions (Table 5).

**Table 5:** Cerebral autoregulation during spontaneous breathing.

		Supine	Seated	Standing
**Gain, %/mmHg**				
*VLF*	**men**	1.43 ± 0.52	1.21 ± 0.30	1.35 ± 0.38
	**women**	1.52 ± 0.55	1.32 ± 0.37	1.43 ± 0.38
*LF*	**men**	1.65 ± 0.35	1.76 ± 0.20	1.90 ± 0.50
	**women**	1.70 ± 0.37	1.64 ± 0.43	1.85 ± 0.33
**Phase, degrees**				
*VLF*	**men**	51.0 ± 16.6	56.1 ± 20.9	58.6 ± 25.4
	**women**	67.7 ± 13.9*	54.1 ± 23.5	58.9 ± 21.9
*LF*	**men**	36.9 ± 12.9	35.8 ± 15.8	36.0 ± 16.5
	**women**	30.6 ± 19.4	38.3 ± 17.2	27.6 ± 8.2
**Coherence**				
*VLF*	**men**	0.40 ± 0.17	0.50 ± 0.21	0.47 ± 0.17
	**women**	0.30 ± 0.16	0.38 ± 0.13	0.39 ± 0.15
*LF*	**men**	0.58 ± 0.15	0.75 ± 0.11†	0.79 ± 0.10†
	**women**	0.47 ± 0.22	0.62 ± 0.22†	0.75 ± 0.09†
**MAP Power, mmHg**^**2**^				
*VLF*	**men**	4.3 ± 2.9	5.7 ± 3.1	5.8 ± 2.7
	**women**	2.4 ± 1.8	3.2 ± 2.0*	3.2 ± 1.7*
*LF*	**men**	1.7 ± 1.1	4.1 ± 2.5†	5.3 ± 4.0†
	**women**	1.0 ± 1.0	2.1 ± 1.2†	3.6 ± 2.2†
**MCAv Power, %**^**2**^				
*VLF*	**men**	15.6 ± 10.8	13.7 ± 13.6	18.3 ± 13.9
	**women**	11.6 ± 10.2	12.5 ± 9.2	15.4 ± 11.2
*LF*	**men**	6.0 ± 3.7	16.6 ± 13.8†	27.2 ± 36.3†
	**women**	3.7 ± 2.0	7.3 ± 4.2†	14.7 ± 11.3†
**MAP, mmHg**				
	**men**	79.1 ± 8.5	81.2 ± 9.9	79.2 ± 8.7
	**women**	73.1 ± 6.2	76.4 ± 8.3	76.4 ± 9.8
**MCAv, cm/s**				
	**men**	70.1 ± 15.6	63.8 ± 14.0†	62.0 ± 16.0†
	**women**	82.5 ± 17.7	77.8 ± 17.8*†	73.3 ± 16.0†
**MCAv, %**				
	**men**	100	91.9 ± 13.3†	88.6 ± 12.4†
	**women**	100	94.3 ± 4.8†	89.3 ± 7.9†
**MCA CPP, mmHg**				
	**men**	79.1 ± 8.5	53.8 ± 12.3†	51.8 ± 11.0†
	**women**	73.1 ± 6.2	48.7 ± 11.5†	48.7 ± 12.2†
**MCA Cerebrovascular Resistance, mmHg/cm/s**				
	**men**	1.18 ± 0.28	0.88 ± 0.26†	0.89 ± 0.29†
	**women**	0.93 ± 0.23	0.65 ± 0.18*†	0.68 ± 0.18†
**End-tidal CO**_**2**_**, mmHg**				
	**men**	40.1 ± 3.1	37.5 ± 4.0	36.0 ± 4.2†‡
	**women**	37.3 ± 2.9	36.3 ± 3.0	35.1 ± 3.2†‡

Values are means ± SD. VLF, very low frequency range (0.02–0.07 Hz); LF, low frequency range (0.07–0.20 Hz); MAP, mean arterial pressure; MCAv, middle cerebral artery velocity; CPP, cerebral perfusion pressure; MCA, middle cerebral artery; women *n =* 10, men *n =* 11, with the exception of gain and phase in the very low frequency range when supine (women *n =* 7, men *n* = 11).

* main effect of sex; *P <* 0.05 vs men;

^†^ *P <* 0.0167 vs. supine;

^‡^ *P <* 0.0167 vs seated.

There were no sex differences in cerebral autoregulatory measures in both the VLF and LF ranges with the exception that women had significantly higher phase in the VLF range when supine (*P* = 0.027; Table 5).

## Discussion

There are two main findings from this study: 1) Posture affects both cerebrovascular CO_2_ reactivity and cerebral autoregulation, independent of sex; and 2) Both men and women have similar cerebrovascular CO_2_ reactivity and similar cerebral autoregulation during spontaneous and slow-paced breathing.

### Effect of body posture on cerebrovascular CO_2_ reactivity

We found improved cerebrovascular CO_2_ reactivity in the supine posture. Our results are similar to those of Regan et al. [14]. In that study, they found increased reactivity when supine compared with seated, but it did not reach statistical significance, likely because of the small sample size (*n =* 9). Mayberg et al. [9] found significantly increased absolute reactivity and not relative reactivity, which is in contrast to the present study, which found improved reactivity when using relative changes in cerebral flow velocity. Additionally, our results are in contrast to those of Tymko et al. [13], which found no differences in reactivity between supine and upright tilt. It is possible that hyperoxic rebreathing conditions may play a role in the discrepant findings between the current study and study by Regan et al. [14] compared to the findings by Tymko et al. [13] and Mayberg et al. [9]. Since hyperoxia would cause a cerebral vasoconstriction [26] that may blunt the CO_2_ mediated vasodilation, this may have obscured differences found with normoxic gas mixtures.

It is possible that improved cerebrovascular CO_2_ reactivity when supine may be because of differences in vascular state. In upright postures, there is lower pressure at the brain [6], and the cerebral vessels must dilate (i.e., lower vascular resistance) in response to the lower pressure. It is possible that there is greater vasodilatory reserve when supine because the vessels begin in a more constricted state. However, we would expect lower vasoconstrictive reserve when supine (i.e. decreased hypocapnic slope), but we did not observe this. It is possible that underlying vascular state can affect the ability to dilate whereas the ability to constrict may be more robust and unaffected by initial vascular state.

It is also possible that the vestibular system played a role in the postural effect on cerebrovascular CO_2_ reactivity. The vestibular system can affect cerebral flow velocity and cerebrovascular resistance when upright, likely an adaptive mechanism to help dilate the cerebral vessels [27]. However, given that we observed greater vasodilation (i.e., hypercapnic slope) when supine when there is no hydrostatic gradient, it seems unlikely the vestibular system had a significant impact on our findings.

### Effect of body posture on cerebral autoregulation during spontaneous breathing

We did not find any significant changes in autoregulation during spontaneous breathing across body postures, which is consistent with most prior research, [10–12] but not all research, including MCA/ACA autoregulatory index [7] and rate of regulation in the vertebral artery [8], which may represent different autoregulatory mechanisms compared with transfer function measures of autoregulation [28]. The lack of changes in gain and phase in our study are similar to prior studies [10–12], suggesting that autoregulation is relatively unaffected by body posture during spontaneous breathing.

### Effect of body posture on cerebral autoregulation during slow breathing

During slow breathing we found significantly improved autoregulation (i.e. lower gain, higher phase, and lower coherence) when supine compared with both seated and standing postures. One explanation for improved autoregulation when supine could be because of reduced spectral power of mean arterial pressure and reduced pressure oscillations challenging the autoregulatory system. The cerebral vasculature may not have been challenged as much because there was a lower driving stimulus, i.e. pressure changes. However, there was also significantly lower mean amplitude of oscillations in middle cerebral artery velocity, and the magnitude of difference between supine vs. seated and standing were greater in cerebral flow velocity than mean arterial pressure. The greater difference between the postures in middle cerebral flow velocity compared to mean arterial pressure amplitude of oscillations is consistent with lower gain when supine. We also must consider the possibility that the increased variability in end-tidal CO_2_ during slow breathing may have affected the autoregulatory measures. However, the variability (Table 4) was not significantly different across the three postures, so end-tidal CO_2_ changes across the slow breathing cycles were unlikely to affect the postural results. Increased vasoconstriction improves autoregulation [29], and cerebrovascular resistance was significantly higher when supine, which could have caused the improved autoregulation. However, cerebrovascular resistance was also higher when supine during spontaneous breathing, but we did not find improved autoregulation in that condition, making it unlikely that higher vascular resistance was the sole driver of the improved autoregulation. Another possible cause of improved autoregulation when supine that must be considered is the role of the vestibular system. However, vestibular effects on the cerebrovascular response would likely be related to changes in cerebrovascular resistance induced through vestibular pathways. As we discussed previously, differences in resistance do not appear to explain differences in autoregulation, suggesting vestibular inputs are unlikely to play a central role. Therefore it remains unclear why autoregulation was improved during slow breathing while supine. Further research is necessary to confirm this finding and elucidate possible mechanisms.

### Sex differences in cerebrovascular CO_2_ reactivity

Similar to previous studies [16, 30, 31], but in contrast to other studies [15, 32–35], we did not find improved cerebrovascular reactivity to hypercapnia in women. The discrepancies between studies cannot be attributed to the type of vasoactive stimuli, because both groups of studies used similar types of stimuli to induce hypercapnia. The inconsistent findings between studies may be due to small sample sizes, individual variability in cerebrovascular reactivity, or overlapping reactivity between healthy men and women.

### Sex differences in cerebral autoregulation

Overall, we did not find any significant sex differences in cerebral autoregulation regardless of posture, indicated by no differences in gain, phase or coherence in the low frequency (LF) range. In the supine posture, we found significantly increased phase in the very low frequency (VLF) range in women, but this was not associated with lower gain or coherence. We also found significantly increased coherence in women in the LF range when standing during slow-breathing, but there were no changes in gain or phase or in the LF range, making it unlikely that differences in coherence had a meaningful impact on cerebral autoregulation. Increased coherence may alternatively indicate increased linearity or increased signal-to-noise ratio between blood pressure and cerebral flow velocity [24].

Assuming our interpretation is accurate that men and women have similar autoregulation, our results are in agreement with those of other groups, which found no differences in autoregulation assessed by autoregulatory index [19, 36] or the slope between blood pressure and middle cerebral flow velocity [37]. However, our results are in contrast to results of our lab’s prior studies which found improved autoregulation in women assessed by thigh-cuff maneuvers [7], sit-to-stand maneuvers [15], and squat-to-stand maneuvers [16]. Additionally, our results are in contrast to other studies that found worse autoregulation in women when supine [17], during head-up tilt [38], and in aerobically trained women [39].

### Limitations

Important limitations of our method for measuring cerebrovascular reactivity are that our hypercapnic stimulus was small (5% CO_2_) and our protocol limited hypercapnia and hypocapnia to two minutes. Using a small stimulus and limiting the time of our stimuli to two minutes may not have captured the full cerebrovascular response [14, 40]. Additionally, our reactivity slopes would have been affected by the speed of the cerebrovascular response because we used the entire time course to calculate the slopes, and individuals differ in lung size and CO_2_ respiratory chemoreflex [14, 40, 41]. All of these factors lead to increased variability in our measurement of cerebrovascular reactivity and must be considered when interpreting our results.

Transcranial Doppler assumes the diameter of the insonated cerebral artery remains constant for accurate measures of cerebral blood flow. Recent research suggests the diameter of the MCA changes during CO_2_ manipulation [42–45], but this concern is still debated [46, 47]. The studies that show a significant change in arterial diameter examined increases in end-tidal CO_2_ of ~10 mmHg and decreases of ~15 mmHg [42–44]. In the current study, end-tidal CO_2_ modestly increased ~6.3 mmHg and decreased ~7 mmHg from normocapnia, making it unlikely that diameter significantly changed in our study. However, we acknowledge a recent study [45] observed changes in arterial diameter during modest changes (+7 mmHg and -10 mmHg) in end-tidal CO_2_. However, they induced hypercapnia and hypocapnia for ten minutes and their previous study [44] found that changes in the MCA diameter did not occur until four minutes. Our study specifically limited hypercapnia and hypocapnia to two minutes to account for a potential time delay in arterial diameter changes. However, we must acknowledge the possibility that the MCA diameter might have changed in our study and our velocity could have underestimated flow.

Another limitation to the present study is that we used end-tidal CO_2_ as a surrogate for arterial CO_2_. End-tidal CO_2_ approximates arterial CO_2_ when supine, but slightly overestimates the drop in arterial CO_2_ when upright [48, 49]. Peebles et al. [50] found that end-tidal CO_2_ overestimates arterial CO_2_ during 8% hypercapnia but not 4% hypercapnia or hypocapnia when supine. Since we used mild levels of hypercapnia (5%) it is likely that end-tidal CO_2_ approximately estimated arterial CO_2_ when supine. It is worthy to note that Pebbles et al. [50] did not assess the response in the seated or standing postures. If arterial CO_2_ overestimation when upright is similar across hypercapnic and hypocapnic ranges, then the slope of the response would remain unchanged, and this overestimation would not explain differences in CO_2_ reactivity.

## Conclusions

Body posture affects cerebrovascular CO_2_ reactivity and cerebral autoregulation during slow breathing, but does not appear to affect cerebral autoregulation under spontaneous breathing conditions. Both cerebrovascular reactivity and cerebral autoregulation during slow breathing were improved in the supine posture compared to seated and standing postures, independent of sex. These data highlight the importance of making comparisons within the same body position to ensure there is not a confounding effect of posture.
